# Social determinants of pulmonary tuberculosis in Brazil: an ecological study

**DOI:** 10.1186/s12890-019-0855-1

**Published:** 2019-05-08

**Authors:** Alessandra Isabel Zille, Guilherme Loureiro Werneck, Ronir Raggio Luiz, Marcus Barreto Conde

**Affiliations:** 1 Instituto de Doenças do Tórax da Universidade Federal do Rio de Janeiro, Rio de Janeiro, RJ Brazil; 2 Instituto de Estudos em Saúde Coletiva da Universidade Federal do Rio de Janeiro, Rio de Janeiro, RJ Brazil; 3 Faculdade de Medicina de Petropolis/FASE, Petrópolis, RJ Brazil; 4 Hospital Universitário Clementino Fraga Filho, Cidade Universitária, Rua, Professor Rodolpho Paulo Rocco n° 255, 6° andar, Rio de Janeiro, 21941-913 Brazil

**Keywords:** Tuberculosis, Income, Inequality, Social indicators

## Abstract

**Background:**

Social determinants may influence the incidence and control of tuberculosis (TB). The aim of this study was to evaluate the correlation between social determinants and pulmonary TB (PTB) incidence and treatment outcomes in different regions in Brazil.

**Methods:**

In this ecological study, PTB incidence and treatment outcome rates as well as HIV incidence for all 5560 Brazilian cities as reported to the Brazilian Tuberculosis Program in 2010 were correlated with two social indicators, the Human Development Index (HDI) and Gini Index (GI). Cities were stratified into six groups based on location (metropolitan region or not) and size (small, medium, and large cities), and according to the regions of the country to which they belong. The Spearman correlation coefficient was used to assess the association between variables.

**Results:**

In 2010, 68,729 new PTB cases were reported in Brazil, with an incidence rate of 36 cases per 100,000 inhabitants. Incidence rates and PTB mortality demonstrated a weak negative correlation with HDI and a positive correlation with GI. The correlation between HDI and GI with cure, relapse, and lost to follow-up of treatment greatly varied in the different groups of cities and regions of the country evaluated.

**Conclusions:**

There is a weak correlation between HDI and GI and PTB incidence and mortality rate. However, there is great variation between the HDI and GI and cure, relapse, and lost to follow-up in the different groups of cities and regions of the country. This suggests that for TB determination, these outcome variables might be more related to the quality of healthcare provided by services than to social determinants in the general population.

## Background

Brazil is a member of BRICS (Brazil, Russian Federation, India, China, and South Africa), a group of countries that together represent approximately 50% of tuberculosis (TB) cases worldwide [1]. However, since the 1980s, the incidence rate of pulmonary TB (PTB) in Brazil has decreased, which ranged from 70.4 TB cases/ 100,000 inhabitants in 1982 to 32.4/100,000 in 2016 [2, 3]. Surprisingly, the decrease in the TB incidence rate has not been followed by an improvement in other data related to TB control such as treatment outcomes. In fact, throughout this period, cure rates remained low (approximately 65%), while lost to follow-up and TB mortality persisted at high levels (approximately 13 and 7%, respectively). Moreover, the increase in the number of multi drug resistance tuberculosis (MDR TB) cases has been well documented [4]. In addition, data from a recent study suggest that despite the changes implemented by the Brazilian TB National Program in 2009 (adding ethambutol to the therapeutic regimen and adopting fixed-dose combined pills, inter alia), these rates are deteriorating [5]. One possible reason for the reduction in the TB incidence rate in a setting where other indices of TB control are deteriorating might be improved social and quality of life factors. In fact, while the PTB incidence rate is less than 10 cases per 100,000 inhabitants per year in high-income countries including those in Europe, Canada, the United States, Japan, Australia, and New Zealand, low-income countries such as Mozambique, South Africa, and Zimbabwe present rates above 500/100,000 inhabitants [6]. Data also demonstrate an inverse relationship between TB incidence and Gross Domestic Product (GDP) [7]. An ecological study in 2000 in California demonstrated that a higher per capita income might act as a “protector” factor for communicable diseases like TB [8]. According to Ploubidis (2012), income inequality is a significant predictor of country-level variation in TB rates, with less inequality associated with a lower TB incidence rate and prevalence [9].

Brazil experienced a significant increase in GDP per capita after the stabilization of the economy in the 1990s. The country has also implemented different social policies in recent years to partially reduce absolute poverty and income inequality [10]. However, Brazil is a continental country and still has significant socioeconomic disparity, with a broad variation of GDP and inequality of income distribution among its different regions [11, 12]. A study conducted in Brazil (1998–2006) associated higher TB/HIV incidence rates and areas with lower socioeconomic levels in the city of São Paulo [13]. Another study conducted between 1990 and 2010 among HIV positive and negative TB patients suggested a correlation between the TB/HIV coinfection rate and poverty in Brazil [14]. Furthermore, an ecological cohort conducted between 2000 and 2005 in the city of Porto Alegre demonstrated from the spatial perspective that the mean annual incidence of TB is not homogenously distributed along the city’s territory. Similarly, the non-spatial analysis demonstrated the narrow relation between TB and social determinants, concentrating the highest rates in the lower socioeconomic districts [15]. To our knowledge, no national study to date has evaluated the association between social determinants and TB indices in all Brazilian cities. While existing studies clarify the association between social inequality and the incidence of TB, there is no correlation between social determinants and the main treatment outcomes (lost to follow-up, relapse, cure, mortality). As such, this paper reports a comparative study between social chains and TB incidence rate, cure, lost to follow-up, recurrence, and mortality in Brazil, considering 5560 municipalities in total and separately to evaluate the influence of the heterogeneity of the population groups evaluated.

## Methods

### Study design

#### Ecological study

##### Studied variables

The study was conducted using all 5560 Brazilian cities that existed in 2010 as the unit of analysis. We evaluated all new pulmonary TB (PTB) cases, treatment outcomes (lost to follow-up, cure, mortality), and relapse rates reported to the Brazilian Diseases Information System (SINAN) by city of residence. Extrapulmonary TB cases were excluded from the analysis based on the lack of evidence of its association with social determinants. We used the TB definition as revised by the WHO in 2013 [16]. The proportion of cure, relapses, and lost to follow-up cases among the total number of PTB cases were evaluated for each city in 2010 [17, 18]. Death due to PTB was assessed according to the International Classification of Diseases (ICD A15-A16). Direct standardization by age group was performed in the calculation of mortality rates by city. The standard population was that of the 2010 Brazilian Census [12, 19].

For social determinants, we evaluated the Human Development Index (HDI) and Gini Index (GI). GI is a globally used indicator that enables analyzing the socioeconomic situation of the population, identifying segments that require greater attention from public health policies, as well as education and social protection, among other factors [20, 21]. It measures the degree of income concentration in a particular group, indicating the differences between the incomes of the poorest and richest. Reportedly, the HDI is one determinant closely correlated with TB incidence by the Commission on Social Determinants of Health (CSDH), forming part of the structural determinants of health that generate or reinforce social stratification [22, 23]. HDI uses an arithmetic average of indices related to three dimensions of human development: longevity, education, and income. Numerically, both indices vary from zero to one and have inverse meanings. For the GI, a zero value represents the situation of equality of income distribution, meaning that everyone has the same income. A value near to one means that a few people hold all the wealth [24]. HDI also varies from zero to one. However, the closer to one the value, the higher is the human development [23]. Since the HDI and GI indices are not published annually for each city, this study used the most recent year (2010) for which both indices and official TB data were reported in Brazil.

Because HIV infection might be a confounding variable in the association between TB and social determinants, we also used the HIV rate as a predictor variable. Because the testing rate among TB cases in Brazil is low, we used the HIV rate reported to the Brazilian AIDS Program in each municipality.

##### Characteristics of the population

Brazil is a large country with a heterogeneous population distributed across 5560 cities living in different social and geographical conditions. Therefore, clusters based on characteristics such as geographic distribution (macro-regions), number of inhabitants, rural or metropolitan location, and population density were used to evaluate the correlation between the HDI and GI with TB. Cities were stratified into six groups: small, medium, and large cities, belonging or not to a metropolitan area. Small cities were considered those with fewer than 50,000 people and a population density of less than 80 inhabitants/km^2^. Medium cities were those with populations between 50,000 and 100,000 inhabitants or with a population density above 80 inhabitants/km^2^ (predominating the number of inhabitants in the classification), while large cities were those with a population of more than 100,000 inhabitants, regardless of population density [25]. A metropolitan region was considered a cluster of neighboring municipalities established by law for the planning and execution of public functions of common interest, often featuring a large urbanized region formed by the city core and adjacent cities [12]. The five macro-regions in Brazil are the North (seven states), Northeast (nine states), Southeast (four states), South (three states), and Midwest (three states). The municipalities were also grouped according to the region to which they belong based on the five macro-regions of Brazil: North (seven states), Northeast (nine states), Southeast (four states), South (three states), and Midwest (three states).

##### Statistical analysis

The mean and total number of cases were used for descriptive purposes. The Spearman correlation coefficient (*r*_*s*_) was used as the measure of association to determine the strength of the relationship between variables, which varies from − 1 (perfect negative correlation) to + 1 (perfect positive correlation). A value of *r*_*s*_ = 0 means no correlation between variables. The closer the extreme value of *r*_*s*_ (− 1 or + 1), the greater is the association between variables. The size of the correlation of values different from 1 was interpreted as follows: ≤0.30 as a negligible correlation, 0.30 to 0.50 as low, 0.50 to 0.70 as moderate, and from 0.70 to 0.90 as a high positive correlation [26]. Statistical analyses were processed using the Statistical Package for Social Sciences (SPSS) version 22.0 for Windows software.

##### Data sources

The data used in this study were collected from different information systems according to the variable studied (updated October 2014). Data on PTB new cases, cure, relapse, and lost to follow-up of the treatment were collected from SINAN. Data on PTB mortality were extracted from the Brazilian Mortality Information System (*Sistema de Informação de Mortalidade*, SIM). The number of AIDS cases, GI, and population of each city used to calculate the PTB incidence rates and mortality (data from the 2010 Brazilian Census) were obtained from the Department of Statistics of the Unified Health System (DATASUS). The United Nations Program for Development (UNDP) served as the source for the HDI for each city.

##### Ethical approval

The study was submitted to the Institutional Review Board (IRB) of the Federal University of Rio de Janeiro with the number 281.382 and was approved on May 9, 2013 without further analysis, because it was an ecological study based on public secondary data. The data used for this study are public and can be accessed freely on the electronic addresses of the Brazilian Government [2–4].

## Results

### General data

In 2010, Brazil housed 190,731,964 inhabitants in 5560 cities. The heterogeneity in population distribution is presented in Table 1. It is evident that 41% (78,483,169/190,731,964 inhabitants) of the country’s population was concentrated in 154 large metropolitan cities. In 31.2% (1733/5560) of the cities, no new PTB cases were reported in 2010, and of these, 88.1% were small cities not located in a metropolitan area (1528/1733) (Table 1).

**Table 1:** Pulmonary tuberculosis and AIDS data and the HDI and GI in different groups of cities in Brazil

Variable	Country		Groups of cities											
			Metropolitan area						No metropolitan area					
Population group	Brazil		Small		Medium		Large		Small		Medium		Large	
	HDI^a^	GI^a^	HDI^a^	GI^a^	HDI^a^	GI^a^	HDI^a^	GI^a^	HDI^a^	GI^a^	HDI^a^	GI^a^	HDI^a^	GI^a^
	0.65	0.50	0.67	0.47	0.69	0.48	0.74	0.51	0.64	0.50	0.66	0.51	0.73	0.53
Inhabitants	190,731,964		3,998,698		8,783,630		78,483,169		48,579,389		24,634,920		26,252,158	
PTB new cases (N)	68,729		851		3316		40,801		10,113		6271		7377	
PTB incidence rate	36		21.2		37.7		51.9		20.8		25.4		28.1	
Cities with no new PTB case(s)	1733		134		17		1		1528		53		0	
Cure (total n of cases)	49,585		635		2442		28,331		7760		4743		5674	
Cure (%)	69		43.3		64.7		72		45.8		65.2		74.7	
Lost to follow-up (N)	9052		79		360		6492		748		607		766	
Lost to follow-up (%)	12		4.1		8.9		11.8		4.2		7.4		9.2	
Relapse (N)	4827		65		287		2925		704		437		409	
Relapse (%)	7		4.3		6.7		6.5		4		6.2		5.4	
AIDS (N)	37,331		460		1702		22,782		3723		3215		5449	
AIDS incidence rate	19.5		11.5		19.3		29		7.6		13		20.7	
Death due to PTB (N)	4235		45		186		2402		757		412		433	
Death due to PTB Incidence rate^b^	1.5		0.91		2.28		3.68		1.4		1.66		1.78	

Incidence rates per 100,000 population

Small Cities < 50,000 inhabitants and density < 80 inhabitants/km^2^, medium cities population between 50,000 and 100,000 or density > 80 inhabitants/km^2^, large cities population > 100,000 inhabitants. The variables cure, relapse, and lost to follow-up are provided as an average of percentages in the groups of cities

*AIDS* Acquired Immunodeficiency Syndrome, *HDI* Human Development Index, *GI* Gini Index

^a^The HDI and GI show the average by population group. ^b^Mortality rate due to PTBwas calculated by direct standardization

In 2010, 71,919 PTB cases were reported in Brazil, of which 95.6% (68,729/71,919) were new cases corresponding to an incidence rate of 36 per 100,000 inhabitants. The proportion of those cured was 69% (49,585/71,919), of relapse 7% (4827/71,919), of lost to follow-up 12% (9052/71,919), and of deaths due to PTB 6% (4235/71,919). The average standardized mortality rate in 2010 was 1.5 per 100,000 inhabitants. In total, 37,331 new AIDS cases were reported in the general population, corresponding to an incidence rate of 19.5 per 100,000 inhabitants (Table 1). In Brazil (2010), the HDI average was 0.65 and GI 0.50.

Table 2 shows that the largest number of new PTB cases occurred in the Southeast region (30,863 PTB new cases), but the highest average incidence rate was in the Southern region (26.9 cases/100,000). The highest average cure proportion was in the Northern region (85.5%). The highest average lost to follow-up proportion was in the Northeast region (5.7), and the lowest in the South (4.2).

**Table 2:** Pulmonary tuberculosis and AIDS data and the HDI and GI in five macro-regions of Brazil

Variable	Brazilian regions									
	North		Northeast		Southeast		South		Midwest	
	449 cities		1792 cities		1668 cities		1188 cities		463 cities	
	HDI	GI	HDI	GI	HDI	GI	HDI	GI	HDI	GI
	0.60	0.57	0.59	0.53	0.69	0.47	0.71	0.46	0.68	0,50
Inhabitants	15,864,454		53,071,442		80,364,410		27,386,891		14,044,767	
PTB cases (N)	7167		20,889		31,733		8979		3151	
PTB new cases (N)	6791		19,623		30,863		8459		2993	
PTB incidence rate	23.7		21.9		21.9		26.9		18.2	
Cities with no new PTB cases	106		380		542		535		170	
Cure (total n of cases)	5234		13,925		22,219		6089		2118	
Cure(%)	85.5		55.3		50.7		40.6		44.5	
Lost to follow-up (N)	798		2516		4240		1169		329	
Lost to follow-up(%)	11.7		12.8		13.7		13.8		10.9	
Relapse (N)	339		1385		2145		791		167	
Relapse (%)	2.9		5.1		4.3		4.7		3.2	
AIDS incidence rate	8.8		6.2		10.3		11.3		9.5	
Death due to PTB rate (N)	330		1247		1171		1133		354	
Death due to PTB rate	1.5		1.9		1.5		0.9		1.8	

Mortality rate due to PTBwas calculated by direct standardization. Incidence rates per 100,000 population. The variables cure, relapse, and lost to follow-up are given as an average of percentages in the regions

*AIDS* Acquired Immunodeficiency Syndrome, *HDI* Human Development Index, *GI* Gini INdex

There was a higher proportion of relapse in the Northeast region (5.1/100,000). The highest rates of AIDS incidence were in the South (11.3/100,000) and Southeast (10.3/100,000), while the highest rate of PTB mortality was in the Northeast region (1.9/100,000) (Table 2).

### Correlation between variables

Table 3 shows that the PTB incidence rate is negatively correlated with HDI (meaning an inverse association between these two variables: the higher the HDI, the lower the TB incidence rate) in all groups of cities. The correlation was not statistically significant in medium cities (metropolitan and non-metropolitan) or small cities located in a metropolitan area. In addition, the correlation was negligible (≤0.30) in all groups except in large/non-metropolitan groups of cities (*r*_*s*_ = − 0.42). PTB incidence rates correlated positively with the GI in all groups (meaning a direct association between these two variables: the higher the GI, the higher the TB incidence rate), and the correlation was statistically significant in all groups, except large cities located in a metropolitan area. Again, the correlation was negligible (≤0.30) in all groups except in large/non-metropolitan groups of cities (*r*_*s*_ = 0.44).

**Table 3:** Correlation coefficients between rates related to PTB and HDI/GI in cities classified by size and population aggregation

Variable	Country		Groups of cities											
			Metropolitan area						No metropolitan area					
Population group	Brazil		Small		Medium		Large		Small		Medium		Large	
	HDI	GI	HDI	GI	HDI	GI	HDI	GI	HDI	GI	HDI	GI	HDI	GI
PTB new cases	−0.05^b^	0.18^b^	−0.01	0.15^b^	−0.07	0.16^a^	−0.25^b^	0.13	−0.16^b^	0.17^b^	−0.03	0.15^b^	−0.42^b^	0.44^b^
Cure	−0.02	0.13^b^	0.05	0.04	0.17^a^	−0.04	0.28^b^	−0.09	−0.11^b^	0.16^b^	0.11^b^	−0.03	0.06	−0.05
Lost to follow-up	0.09^b^	0.14^b^	−0.03	0.17^b^	− 0.01	− 0.02	− 0.13	0.18	− 0.07^b^	0.12^b^	0.08	0.12^b^	0.13	−0.01
Relapse	0.1^b^	0.11^b^	−0.02	0.13^a^	0.11	0.09	0.09	−0.08	−0.06^b^	0.09^b^	0.14^b^	0.05	−0.02	−0.21^a^
AIDS	0.27^b^	0.03^a^	0.1^a^	0.04	0.37^b^	−0.22^a^	0.18^a^	0.07	0.14^b^	0.04^b^	0.34^b^	−0.07	0.26^b^	0.04
Death due to PTB	0.02^a^	0.16^b^	−0.08	0.14^a^	−0.14^a^	0.22^b^	−0.44^b^	0.06	−0.11^b^	0.14^b^	−0.01	0.18^b^	−0.35^b^	0.34^b^

Brazilian population in 2010: 190,731,964 inhabitants. Incidence rate per 100,000population. Small Cities< 50,000inhabitantsand density < 80 inhabitants/km^2^, medium cities population between 50,000 and 100,000 or density > 80 inhabitants/km^2^, large cities population > 100,000inhabitants

Coefficients were calculated between the HDI/GI and % of cure, % lost to follow-up, % relapse, PTB incidence rate, AIDS incidence rate, and rate of deaths due to PTB (calculated by direct standardization)

*AIDS* Acquired Immunodeficiency Syndrome, *HDI* Human Development Index, *GI* Gini Index

^a^Significant correlation at the 0.05level (2 tailed), ^b^Significant correlation at the 0.01 level (2 tailed)

The cure rate was positively associated with HDI in most groups (the higher the HDI, the higher the cure rate), except in small non-metropolitan cities. However, the correlation was negligible in all groups and statistically significant in just two groups (Table 3). The correlation between the GI and cure rate did not present a single pattern, and was positive (the higher the inequality, the higher the cure rate) in three groups and negative (the lower the GI or inequality, the higher the cure rate) in four groups. In all groups, the correlation between the GI and cure rate was negligible (≤0.30).

Lost to follow-up and HDI were positively correlated (the higher the HDI, the higher the lost to follow-up) in four groups and negatively correlated (the higher the HDI, the lower the lost to follow-up) in three groups (Table 3). Lost to follow-up and the GI were positively correlated (the higher the GI or inequality, the higher the lost to follow-up) in five groups (in which four were statistically significant) and negatively correlated (the higher the GI or inequality, the lower the lost to follow-up) in two groups (Table 3). However, in all groups, the correlation between the HDI and GI with lost to follow-up was negligible (≤0.30).

Relapse and HDI correlated (negligibly) with a mixed pattern, and the correlation was significant and positive in non-metropolitan medium cities (*r*_*s*_ = 0.14) and negative in small and non-metropolitan cities (*r*_*s*_ = − 0.06) (Table 3). The correlation between the GI and relapse was positive in all five regions and in all cities except large ones (metropolitan and non-metropolitan).

Death and HDI were negatively correlated (negligibly) in all groups of cities (Table 3) and positively correlated (Table 4) in five regions of Brazil. The correlation between the GI and death was positive in all cities and all five regions. Table 3 also shows a positive and significant correlation between the AIDS incidence rate and HDI in all groups. It was more evident in medium cities (metropolitan and non-metropolitan) and agreed with the national correlation, which was also positive (*r*_*s*_ = 0.27).

**Table 4:** Correlation coefficients between rates related to PTB and the HDI/GI in Brazil’sfive macro-regions

Variable	Brazilian regions									
	North		Northeast		Southeast		South		Midwest	
	449 cities		1792 cities		1668 cities		1188 cities		463 cities	
	HDI	GI	HDI	GI	HDI	GI	HDI	GI	HDI	GI
Incidence rate of TB	−0.11	0.22	0.18	0.08	0.13	0.15	0.05	0.44	−0.02	0.25
Cure	−0.14	0.2	0.07	0.06	0.14	0.12	0.05	0.03	0.04	0.24
Lost to follow-up	−0.01	0.09	0.20	0.08	0.30	0.21	0.14	0.05	0.14	0.15
Relapse	−0.05	0.01	0.24	0.08	0.27	0.18	0.15	0.68	0.15	0.2
AIDS	0.2	−0.02	0.27	0.09	0.38	0.1	0.23	0.1	0.17	0.1
Death dueto PTB	0.07	0.01	0.16	0.09	0.23	0.2	0.11	0.11	0.1	0.21

Death due to PTB rate calculated by direct standardization. Incidence rates per 100,000 population. Coefficients were calculated between the HDI/GI and % of cure, % lost to follow-up, % relapse, PTB incidence rate, death due to PTB rate, and AIDS incidence rate

*AIDS* Acquired Immunodeficiency Syndrome, *HDI* Human Development Index, *GI* Gini Index

Table 4 shows a negative correlation between HDI for the majority of variables studied (incidence, cure, lost to follow-up, and relapse) in the North region (the higher the HDI, the lower the rate of the variable evaluated). It also demonstrated a positive correlation between the HDI for most variables (the higher the HDI, the higher the rate of the variable evaluated) in the remaining four regions (Northeast, Southeast, South, and Midwest). The correlation between the GI and all variables studied was positive (the higher the GI or inequality, the higher the rate of the variable evaluated) in all five regions with only one exception (Table 4). Again, with few exceptions, the correlation between the HDI and GI with incidence rate, cure, lost to follow-up, relapse, AIDS, and death due to TB was negligible (≤0.30) in all regions evaluated.

## Discussion

To our knowledge, this is the first study to evaluate the association between the HDI and GI with the incidence rates of PTB, cure rate, lost to follow-up, recurrence, and death due to TB, as well as AIDS in Brazil city by city according to different population groups and regions of the country (including all 5560 cities). Our data weakly correlated the HDI and GI with the incidence and mortality rate of PTB, and demonstrated great variation between the HDI and GI and the cure, relapse, and lost to follow-up in different groups of cities and regions of Brazil.

The present study has numerous limitations. The use of different databases with different management routines can be considered a limitation. Another limitation was due to data unavailability and paucity, i.e., other variables shown to be related to TB could not be included, such as gender, alcohol consumption, drug abuse, diabetes prevalence, nutritional status, and supervised treatment. Regardless of these limitations, the analysis of risk variation in ecological studies, although considered simple for understanding social determinants in health conditions, is considered important in terms of generating new hypotheses, as in the case of the socioeconomic condition of the population groups, by explaining the health conditions of these groups.

Studies that seek to associate the occurrence of TB with socioeconomic indicators (representatives of living conditions) do not always find concordant results. Such divergence may be related to the level of territorial aggregation of the data, as well as the particular characteristics inherent in the populations under study. Although the correlation was negligible (< 0.30) in the majority of cities, it clearly demonstrated that a better educational level and higher income (as demonstrated by a higher HDI), as well as a lower level of inequality (as demonstrated for a lower GI), were associated with a lower incidence rate and mortality due to PTB. These results accord with the findings of Janssens (2008) and Dye (2009), which demonstrated an inverse relationship between TB incidence and increase in GDP, HDI, access to basic sanitation, and low infant mortality in 134 countries on five continents [7, 27]. Furthermore, Castañeda-Hernández et al. (2013) related HDI to TB incidence in 165 countries, and found higher TB rates in countries with lower HDI values [28]. A study in southern India confirmed a significantly higher prevalence of TB among people with a low standard of living index than among those with a medium or high standard of living [29]. An ecological study conducted in Porto Alegre (Brazil) indicated extremely high TB incidence rates in the poverty-stricken areas of cities, reinforcing the importance of inequality in income distribution in health determinants [15]. Results from Pelissari (2017) support a positive association between the TB incidence rate and income inequality distribution as well as an inverse association of this disease with mean per capita household income [30]. A study analyzing the overall 27 European Union member countries as well as Norway and Iceland using a public wealth index (which divides a nation’s economic wealth by its level of social cohesion) and the inequality of income distribution ratio showed an inverse relationship between Proximity Warning Indicator (PWI) scores and TB rates [31]. In this context, it is important to note that the GI values in the different cities studied ranged from 0.47 to 0.53, and according to the 2005 United Nations report, only Namibia (GI = 0.707, 1993), Sierra Leone (GI = 0.629, 1989), Paraguay (GI = 0.578, 2002), Chile (GI = 0.571, 2000), and Argentina (GI = 0.522, 2001) had a GI higher than Brazil.

At the ecological level, it was possible to verify that indicators related to income, schooling, and population density are associated with TB at the different levels of spatial aggregation [32]. Barr et al. associated a 10% increase in the proportion of families living below the poverty line with a 33% increase in the incidence of TB in neighborhoods in New York from 1984 to 1992 [33]. Magnati et al. found that a 1% increase in the proportion of households with more than one person per room represented a 12% increase in the average TB reporting rate for London districts between 1982 and 1991 [34]. Despite the relationship between TB and socioeconomic indicators, their association seems to be influenced by the spatial aggregation level of the data and by particular characteristics of the geographic areas under study.

Castañeda-Hernández et al. (2013) demonstrated that countries with an HDI of less than 0.60 had TB incidence rates above 250 cases/100,000 inhabitants and those with an HDI above 0.90 had a rate below 10 cases/100,000 inhabitants. Based on this information, a PTB incidence rate higher than 36 cases/100,000 inhabitants would be expected in a country with an HDI of 0.65 or 0.50, such as Brazil [28]. A recent study indicated that rates of cure, lost to follow-up, TB mortality, and resistance to TB drugs in Brazil remain far from the rates recommended by the WHO or have perhaps even deteriorated over the past 20 years [5]. However, during this same period, the TB incidence rate decreased. The deterioration of TB control rates (cure, lost to follow-up, TB drug resistance, mortality) and the negligible correlation between HDI and GI with the PTB incidence rate suggest the possibility of PTB under-reporting in Brazil.

The correlations between the social determinants studied (HDI and GI) and variables lost to follow-up and TB relapse were negligible, varying from a positive to negative correlation in different cities and regions and making difficult any definite inference about the association with these variables in this population. Possibly, these two variables are more related to the quality of healthcare services in each region than to social determinants of health in the general population.

Our findings did not confirm other studies conducted in Brazilian cities such as that by Oliveira (2000) in Campinas, which associated treatment lost to follow-up in illiterate or poorly educated subjects [35]. The study findings demonstrated a negative and positive correlation between death from TB and the HDI and GI, respectively, when analyzed as a national average. When we separated the cities into similar groups, the correlation with HDI became negative, demonstrating how heterogeneity can conceal differences. Similar data were found in countries with lower income inequality such as Norway [36]. In addition, the PTB mortality rate was higher than the national rate and higher than other studies conducted in Brazil in the group of larger cities [37]. A review article demonstrated that low-income and private areas in large cities in developed countries have the highest TB incidence and mortality rates [38]. Our findings are similar to those of two other studies that inversely associated TB mortality and HDI [28, 39, 40]. In a similar study, a direct association was found between TB mortality and the Robin Hood index (proportion of income that should be withdrawn from the rich and transferred to the poor to obtain an equitable distribution), average income ratio between the richest 10% and poorest 40%, and the proportion of heads of household with average incomes between one and two minimum wages [40]. Note that poverty is a heterogeneous phenomenon that varies greatly in terms of type and magnitude between countries, regions, or neighborhoods. Socioeconomic and epidemiological indicators do not act in isolation, but according to their own conjuncture characteristics that should be considered in the analyses [41].

Concerning AIDS incidence, there is a higher concentration of cases in metropolitan areas in the Southeast macro-region (most populous region), and previous data demonstrated an unequal distribution of TB/HIV coinfection as well as isolated TB [42]. As in this study, in the United States, the HIV and AIDS epidemic is not evenly distributed across states and regions, but generally concentrated in urban areas. Higher rates of persons diagnosed with HIV or AIDS occur among those usually living in major metropolitan areas [43]. The incidence of AIDS has been shown to be more frequent in large cities, and data indicate a significant positive correlation with HDI and negative correlation with the GI. This finding suggests that in 2010, AIDS was more prevalent among persons with better social conditions, which may explain why Brazil had the lowest impact of AIDS on TB than other countries [42]. Some studies challenged the idea that poverty induces the HIV epidemic, demonstrating that HIV prevalence can be higher in wealthier populations [44, 45]. However, it should be emphasized that in Brazil, only a small proportion of PTB patients are tested for HIV [46]. The epidemiological association of TB with AIDS represents a major challenge considering the difficulties in the organization of the control actions of the two diseases, which are performed by separate and disjointed control policies [47].

The GI is calculated from the Lorenz curve, which may underestimate the real value of the inequality if the richest group uses the income more efficiently than the poorest group. In this way, a GI value can have different meanings. Thus, while a GI of 0.50—such as for Brazil—could mean that 50% of the population has no income and the remaining 50% share the income equally, it could also mean that 75% of the population shares 25% of the income, and the remaining 25% shares 75% of the income [48]. Moreover, in this study, the AIDS incidence rate in the country was evaluated, not TB/AIDS coinfection rates. The databases (SINAN and DATASUS) are not unified and the HIV testing rate among TB cases in Brazil is very low. It is emphasized that data were only collected for patients diagnosed with AIDS, not for HIV-positive patients.

## Conclusion

The correlation between the HDI and GI with the results of cure, relapse and loss of follow-up of the treatment varied greatly in the different groups of cities and regions of the country evaluated. We conclude that these variables may be more related to the quality of care provided by services than to social determinants in the general population. In addition, the decrease in the incidence of TBP followed by deterioration of TB control rates (cure, loss of follow-up, drug resistance, mortality) in the last 20 years and the negligible correlation of the HDI and GI with the incidence rate of PTB may suggest the possibility of PTB sub-notification in Brazil.

## Abbreviations

AIDS: Acquired Immune Deficiency Syndrome
CSDH: Commission on Social Determinants of Health
DATASUS: Department of the Unified Health System
GDP: Gross Domestic Product
GI: Gini Index
HDI: Human Development Index
HIV: Human Immunodeficiency Virus
IBGE: Brazilian Geographic and Statistics Institute
ICD: International Classification of Diseases
NTP: National Tuberculosis Control Program
PTB: Pulmonary Tuberculosis
SD: Standard Deviation
SIM: Mortality Information System
SINAN: Notifiable Diseases Information System
TB: Tuberculosis
UNDP: United Nations Program for Development
US: United States
WHO: World Health Organization
